# Distinct Patterns of Urban-Rural and Sex Disparities in Children's BMI Trajectories From 2013 to 2018

**DOI:** 10.3389/fpubh.2021.689021

**Published:** 2021-07-22

**Authors:** Yunping Zhou, Pengli Yu, Yanqing Zhang, Tao Wang, Aimin Wang

**Affiliations:** ^1^School of Nursing, Qingdao University, Qingdao, China; ^2^Zibo Center for Disease Control and Prevention, Zibo, China; ^3^School of Public Health, Qingdao University, Qingdao, China

**Keywords:** children, body mass index, trajectory, sex, region

## Abstract

**Background:** To identify distinct trajectories of body mass index (BMI) in a large sample of Chinese children by urban-rural and sex disparities.

**Methods:** Data for this study were obtained from the National Surveys on Chinese Students' Constitution and Health among 16,060 children aged 6–11 years. Weight and height data were used to calculate BMI. Group-based trajectory modeling (GBTM) was used to identify distinct BMI trajectories.

**Results:** Seven distinct trajectories were identified, “sustained healthy weight” (46.01%), “sustained obesity” (17.26%), “sustained underweight” (4.50%), “obesity to overweight” (6.45%), “obesity to healthy weight” (11.75%), “healthy weight to overweight” (8.67%), and “healthy weight to obesity” (5.36%). The proportions of “sustained obesity,” “healthy weight to obesity,” and “healthy weight to overweight” trajectories were much higher among boys compared with girls (*P* < 0.001). Meanwhile, children living in rural areas were more represented in the “healthy weight to obesity” trajectory (*P* < 0.001).

**Conclusion:** In this study, the proportions of BMI development trajectories among 6–11-year-old children varied by sex and urban-rural areas, which may require tailored interventions specifically toward these at-risk trajectories.

## Introduction

To our knowledge, the prevalence of childhood obesity has increased rapidly over time and attracts significant attention from many fields. It is reported that the prevalence of obesity among Chinese children aged 7–18 years increased to 7.5% in 2006–2010 (1) and still rose in 2010–2015 (2). Importantly, almost 28.0% of children will be overweight and obese by 2030. Childhood obesity is known to have immediate and long-term unhealthy consequences (3, 4). Body mass index (BMI) trajectories could reflect the potential obesity dynamic changing patterns during the life course (5) and might provide a valuable dimension for consideration. Such research is necessary because it will enhance our understanding to identify the possible risk factors of obesity onset and development during childhood.

Most of the previous reports have shown differences in the prevalence of obesity among children by sex, age group, and urban-rural regions (6). However, they only focused on one time point and ignored the dynamics of childhood obesity. Although a few studies have explored the risk factors and detrimental outcomes associated with children's BMI trajectories (5, 7–9). There is still a paucity of studies exploring the difference of children's BMI trajectories in terms of urban-rural and sex parameters. In China, rapid economic development and dramatic changes to lifestyles and eating habits have led to a substantial difference in the prevalence of obesity among children in recent years (10). Thus, this study aimed to provide current estimates of sex and urban-rural BMI trajectories and dynamic change of obesity status with age in China.

## Methods

### Sampling Design

Data for this study came from the National Surveys on Chinese Students' Constitution and Health 2013–2018 in Zibo city, which is an ongoing nationally representative school-based sample of children followed into adolescents. A sample of 17,175 students (8,910 boys and 8,265 girls) aged 6 years in 2013 participated in this study. After 6 years of follow-up, 16,060 students (8,347 boys and 7,713 girls) completed six measurements of height and weight. Informed consent was provided by a parent or legal guardian for all study participants. The study was approved by the Ethics Committee of the Affiliated Hospital of Qingdao University (project identification code: 20130817). This study was performed in accordance with the ethical standards of the 1964 Declaration of Helsinki and later amendments.

In this research, a stratified multi-stage cluster randomization sampling method was used. In the first stage, Zibo city was divided into 10 municipal districts, each was considered as a stratum. The number of schools in each stratum was determined using the stratified sampling method proportional to size and the number of schools in that stratum. In the second stage, once the sample size in each stratum was determined, the schools within the strata were considered as clusters and the Excel 2016 software was used to cluster two randomly selected elementary schools from each district in order to collect the representative data. In the third stage, all the students aged 6 years in the first grade were invited to participate in this study. The sample size was determined through the use of the following formula and taking into account α = 0.05, π =4% (the rate of the “healthy weight to obesity” trajectory), δ = 0.15π, design effect = 1.5. Considering the possibility of withdrawal at 10%, the final sampling size was 17,175 participants.

$$\begin{array}{l} n=deff\frac{U_{\alpha }^{2}\pi \left(1-\pi \right)}{\delta^{2}} \end{array}$$

### Data Collection

The present study included children with available information on sex, age, region (urban/rural), and data of height and weight. The height and weight were measured annually. All measurements were conducted by a team of trained technicians in each of the 10 districts using the same type of apparatus and following the same procedures. Height was measured using a wall-mounted stadiometer to the nearest 0.1 cm and weight was measured with a standardized scale to the nearest 0.1 kg. Both height and weight were measured twice, and the mean value was recorded. Body mass index (BMI) was calculated using the following formula: BMI = weight (kg)/height^2^ (m^2^). Subjects were defined as being overweight or obese by referring to the age-specific and sex-specific classification criteria for Screening Overweight and Obesity in Chinese Children and Adolescents.

### Statistical Analysis

The group-based trajectory modeling (GBTM) approach implemented in SAS Proc Traj was used to identify trajectory groups between ages 6 and 12 years that shared similar underlying trajectories of body shape (11). Chi-square tests were conducted to estimate the disparities of BMI trajectories by sex and urban-rural areas. All statistical analyses were conducted using SAS V.9.3 (SAS Institute, Cary, North Carolina, USA). The statistical significance level was set to α = 0.05 for all association analyses.

## Results

**Figure 1:**
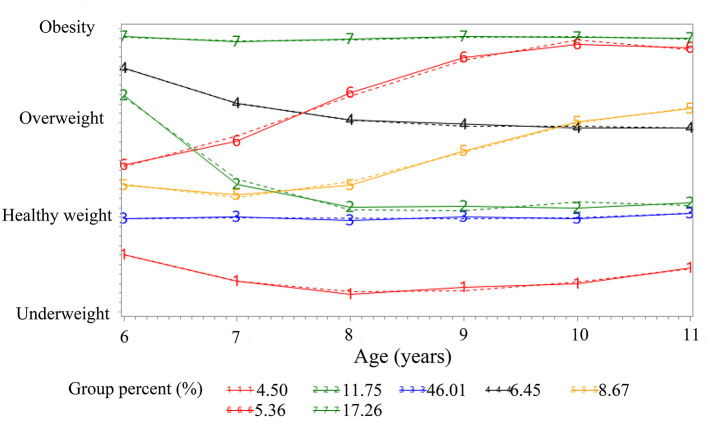
Trajectories of body shape during children from 6 to 11 years. Solid lines represent observed values, and dashed lines represent expected values. Trajectory 1, “sustained underweight” (4.50%); Trajectory 2, “obesity to healthy weight” (11.75%); Trajectory 3, “sustained healthy weight” (46.01%); Trajectory 4, “obesity to overweight” (6.45%); Trajectory 5, “healthy weight to overweight” (8.67%); Trajectory 6, “healthy weight to obesity” (5.36%); Trajectory 7, “sustained obesity” (17.26%).

Using BIC to assess the goodness-of-fit of the competing GBTM models, seven discrete BMI trajectories were identified among the 16,060 participants (Figure 1, Table 1). A total of 4.50, 46.01, and 17.26% followed a trajectory where the average predicted BMI levels remained within underweight (Trajectory 1, “sustained underweight”), healthy weight (Trajectory 3, “sustained healthy weight”), and obesity body shapes (Trajectory 7, “sustained obesity”), respectively. A total of 8.67% of participants maintained a progressive overweight body shape (Trajectory 5, “healthy weight to overweight”), 5.36% followed the trajectory of a progressive obese body shape (Trajectory 6, “healthy weight to obesity”), 6.45% started out as obese then experienced a decrease to an overweight body shape (Trajectory 4, “obesity to overweight”), 11.75% started out as obese then experienced a decrease to a healthy weight body shape (Trajectory 2, “obesity to healthy weight”).

**Table 1:** Participant disparities by sex and urban-rural region for each of the seven different BMI trajectory groups.

**Trajectories**	**Total**	**Sex**		**Region**	
		**Girls**	**Boys**	**Urban**	**Rural**
Sustained underweight (Trajectory 1)	723 (4.50%)	368 (4.77%)	355 (4.25%)	398 (4.54%)	325 (4.45)
Obesity to healthy weight (Trajectory 2)	1,887 (11.75%)	1,069 (13.86%)	818 (9.80%)	1,082 (12.35%)	805 (11.03%)
Sustained healthy weight (Trajectory 3)	7,389 (46.01%)	3,954 (51.26%)	3,435 (41.15%)	3,782 (43.18%)	3,607 (49.40%)
Obesity to overweight (Trajectory 4)	1,036 (6.45%)	484 (6.28%)	552 (6.61%)	675 (7.71%)	361 (4.94%)
Healthy weight to overweight (Trajectory 5)	1,392 (8.67%)	468 (6.07%)	924 (11.07%)	743 (8.48%)	649 (8.89%)
Healthy weight to obesity (Trajectory 6)	861 (5.36%)	321 (4.16%)	540 (6.47%)	416 (4.75%)	445 (6.10%)
Sustained obesity (Trajectory 7)	2,772 (17.26)	1,049 (13.60%)	1,723 (20.64%)	1,663 (18.99%)	1,109 (15.19%)
*P-*value^a^		<0.001		<0.001	

*BMI, body mass index*.

a *P-values from x^2^ tests of independence (comparison of proportions)*.

Disparities were indicated in the proportions and the patterns of the BMI trajectories modeled separately by sex and region (*P* < 0.001) (Table 1). Of note, there were strong associations between children's BMI trajectory assignment and the region and sex of the children. The results showed that boys were more likely to follow the “sustained obesity,” “healthy weight to obesity,” and “healthy weight to overweight” trajectories. Similarly, children living in a rural area were more likely to follow the “healthy weight to obesity” trajectory.

## Discussion

This study revealed that seven body shape trajectories were identified in children aged 6–11 years. Similarly, a systematic review indicated conclusively that the number of children's BMI trajectories often ranged from three to seven (12). According to our data, a large number of children followed the trajectory of “sustained obesity” with the exception of a healthy trajectory, which was consistent with several previous studies (6, 13, 14). Therefore, practitioners and researchers should pay more attention to a decrease in the rate of obesity maintenance in the future.

Our study indicated that sex was a key factor of trajectory assignment. Boys had higher odds of following unhealthy weight trajectories, such as “sustained obesity,” “healthy weight to obesity,” and “healthy weight to overweight” trajectories. Sun et al.'s study showed a similar result that strongly supported our findings (15). Dietary and physical activity behaviors posed a possible explanation for this discrepancy. First, family or social environment and personal perception exerted different impacts on the control of diet for girls but not for boys (16). Girls tended to choose more fad dieting or unhealthy eating behaviors to pursue a slim figure especially during adolescence (17). Furthermore, along with a fast-paced life and widespread use of the Internet, a report of risk behavior monitoring indicated higher odds in the intake of soft drinks and time spent playing computer games for boys than girls, which might contribute to the obesity disparity (18). Accordingly, different health-care recommendations and health resources should be taken into consideration for these at-risk groups in follow-up investigation and intervention.

Our results suggested a complex relationship between region and children's BMI trajectories. The data only showed that children living in rural areas were more likely to follow the trajectory of “healthy weight to obesity,” which might largely be accounted for by the urbanization of rural areas in recent years. Although many previous studies have reported that the prevalence rate of being overweight or obese are higher in children living in urban compared to those living in rural areas (19), a meta-analysis indicated that the difference had decreased over time due to urbanization (20). Indeed, the surveys in rural areas of Shandong province documented that the rate of obese and overweight children was rapidly increasing over time (10, 21). There was a minimal urban-rural difference with regard to healthy diet and physical activity (22). The increasing trend of urbanization was consistent with the rate of obese or overweight children. Consequently, future longitudinal studies in various regions with regard to different eating habits and economic situation are warranted to verify our results.

Although this study provided a novel insight and valuable opportunity to target interventions for children who are overweight and obese, some limitations are still apparent. First, we focused our analyses on modeling the temporal dynamics of BMI change with age. We did not examine potential behavioral or psychosocial factors influencing childhood obesity trajectories which might explain the disparities by sex and urban-rural areas. Addressing this question requires additional covariates which were not available in our analysis and is a topic for future research. Second, GBTM is an exploratory data-driven technique and it is possible that chance relationships in our data will influence trajectory group findings. We were conservative in identifying most likely latent class groups and the trajectories we identified were plausible.

## Conclusion

This study provided a comprehensive estimation on the disparities of body shape trajectories of 6–12-year-old children in a representative sample for sex and urban-rural areas in eastern China. Boys and children living in rural areas were at increased risk of following unhealthy BMI trajectories. The results will provide valuable evidence for the control of being overweight and obese in children. Health professionals and health-care institutions should take different measures in terms of different sex and regions of children.

## Data Availability Statement

The raw data supporting the conclusions of this article will be made available by the authors, without undue reservation.

## Ethics Statement

The studies involving human participants were reviewed and approved by Ethics Committee of the Affiliated Hospital of Qingdao University. Written informed consent to participate in this study was provided by the participants' legal guardian/next of kin.

## Author Contributions

All authors listed have made a substantial, direct and intellectual contribution to the work, and approved it for publication.

## Conflict of Interest

The authors declare that the research was conducted in the absence of any commercial or financial relationships that could be construed as a potential conflict of interest.
